# Development of an algae extract-based culture medium for *Paenibacillus larvae* without animal-derived components

**DOI:** 10.1016/j.mex.2025.103638

**Published:** 2025-09-19

**Authors:** Štěpán Ryba, Adéla Píchová, Petr Mráz, Irena Hoštičková, Michaela Horčičková

**Affiliations:** Faculty of Agriculture and Technology, University of South Bohemia, Branišovská 1760, 370 05 České Budějovice, Czech Republic

**Keywords:** Chlorella, AFB, Cultivation

## Abstract

The cultivation of *Paenibacillus larvae*, the etiological agent of American foulbrood, traditionally relies on media containing animal-derived nutrients. In response to ethical and practical concerns associated with such components, we developed and validated a new culture medium based on an algae extract. The formulation includes *Chlorella* as the principal nutrient source and is supplemented with uric acid and L-tyrosine to support spore germination under conditions resembling the honeybee larval gut. The method includes optimized procedures for dissolving sparingly soluble components and minimizing nutrient degradation. Performance of the new medium was comparable to that of MYPGP and other conventional media in supporting spore germination and vegetative growth of *P. larvae*.

• Eliminates the need for animal-derived components through the use of microalgal nutrients

• Supports robust germination and colony development of *P. Larvae*

• Provides an ethical and cost-efficient medium suitable for diagnostic and research applications

## Graphical abstract

**Figure:**
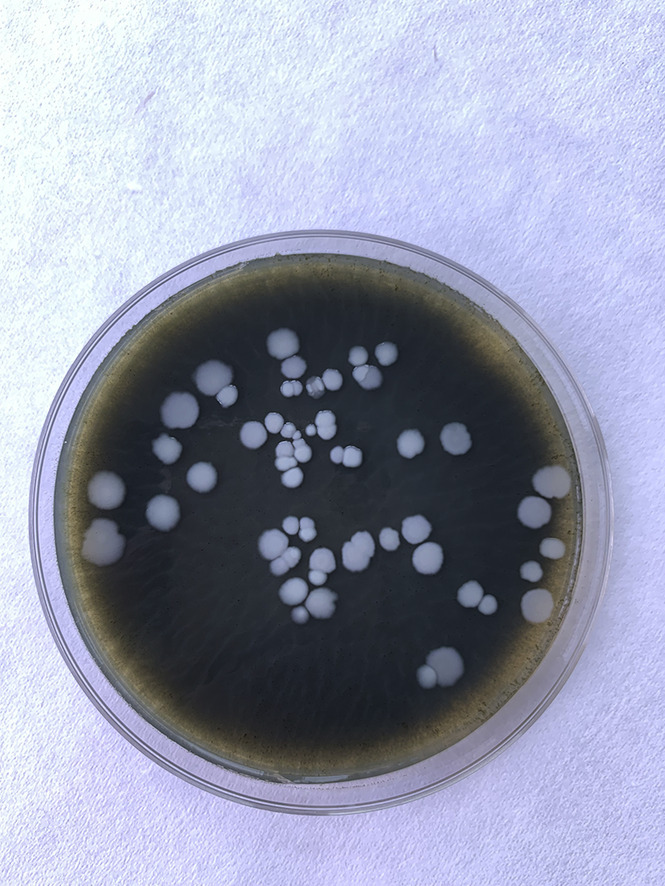

## Specifications table

**Table:** 

**Subject area**	Agricultural and Biological Sciences
**More specific subject area**	**Applied Microbiology and Biotechnology**
**Name of your method**	Algae Extract-Based Culture medium for *Paenibacillus larvae* cultivation
**Name and reference of original method**	None
**Resource availability**	None

## Background

American foulbrood (AFB) is a highly destructive bacterial disease affecting honeybee colonies globally. The causative agent, *Paenibacillus larvae*, is a spore-forming bacterium that infects and kills bee larvae, leading to significant colony losses. Detection of *P. larvae* is achieved through various methods, ranging from PCR to cultivation [1,2]. Effective cultivation and study of *P. larvae* are essential for diagnosing and mitigating AFB outbreaks. Traditionally, the cultivation of *P. larvae* spores has relied heavily on media containing animal-based components, which have served as critical nutrient sources [[3], [4], [5]]. However, the development of new, sustainable, and ethically favorable alternatives to these traditional media is necessary due to concerns about the use of animal products and the challenges posed by the cost and availability of animal-derived components.

One of the most commonly used culture media for *P. larvae* is MYPGP agar, which contains meat peptone, yeast extract, glucose, sodium pyruvate, and potassium permanganate [6,7]. This medium supports the robust growth of *P. larvae* and has been widely adopted in laboratory settings. Other traditional media, such as Columbia sheep blood agar, J-medium, and Brain Heart Infusion (BHI) agar, also provide the rich nutritional environments required for the growth of this pathogen [3]. These media facilitate not only the cultivation of the bacterium but also allow for the observation of haemolytic activity and other diagnostic characteristics [8]. Nevertheless, relying on animal-derived components in these media presents ethical concerns and practical limitations. In response, research efforts have begun exploring alternative formulations that exclude such components without compromising the ability to effectively culture *P. larvae* [9].

*P. larvae* spore germination is crucial for American foulbrood (AFB) infections [10]. Research has shown that spores require l-tyrosine and uric acid to germinate under physiological pH and temperature conditions. Optimal germination occurs near neutral pH (5–7) and temperatures above 35 °C, resembling the honey bee larvae gut environment [11,12]. Our algae extract-based medium was designed for these conditions and enriched with l-tyrosine and uric acid to mimic the larval gut. This plant-based medium effectively supports spore germination at optimal temperatures (35–37 °C).

In controlled trials, this medium performed on par with MYPGP, Columbia agar, and BHI agar, effectively supporting spore germination and vegetative cell proliferation. Its formulation, enriched with algae-derived nutrients [13], obviates the need for animal-derived components and minimizes the risk of contamination by agents such as BSE. Moreover, this innovative medium matches conventional media's efficacy and offers a sustainable, ethically sound, cost-effective alternative for cultivating *P. larvae*, thereby advancing environmentally responsible AFB research.

## Method details

### Materials

Chemicals: All chemicals used in this study were obtained from Sigma-Aldrich (St. Louis, MO, USA).

Dehydrated Algae: Dehydrated Chlorella algae were used as the primary nutrient source in the new medium (Far East Bio-Tec Co., Ltd. Ping-Tung, Taiwan; Product Code: [DB01000201]).

Bacterial strain of *P. larvae*: The bacterial strain of *Paenibacillus larvae* used in this study was obtained from the Czech Collection of Microorganisms, Brno, Czech Republic (CCM 4488). This strain corresponds to the ERIC I genotype.

### New green paenibacillus larvae medium (GPL) preparation

The New Green Paenibacillus larvae medium (GPL) was formulated based on the composition of the established MYPGP medium but incorporating *Chlorella* algae extract as a replacement for animal-derived components. The final composition for a 1-liter batch of GPL medium is detailed in Table 1.

**Table 1:** Composition of the New Green Paenibacillus larvae Medium (GPL) (per 1 Liter).

*Component*	*Amount*	*Approximate Final Concentration*	*Role*
*Agar*	*20 g*	*2% (w/v)*	*Solidifying agent*
*Chlorella algae extract*	*10 g*	*1% (w/v)*	*Primary nutrient source*
*Yeast extract*	*15 g*	*1.5% (w/v)*	*Source of vitamins, amino acids, etc.*
*Potassium phosphate dibasic (K_2_HPO_4_)*	*3 g*	*0.3% (w/v)*	*Buffer, source of phosphorus and potassium*
*Sodium pyruvate*	*1 g*	*0.1% (w/v)*	*Energy source*
*Uric acid*	*∼0.01 g*	*3 mM*	*Spore germination stimulant*
*L-tyrosine*	*∼0.011 g*	*3 mM*	*Spore germination stimulant*
*Glucose*	*2 g*	*0.2% (w/v)*	*Carbon and energy source*
*Distilled water*	*To 1000 ml*	*–*	*Solvent*

The molar mass of uric acid (C_5_H_4_N_4_O_3_) is approximately 168.11 g/mol 1. To prepare a 3 mM solution in 20 ml, the required mass is (0.003 mol/L) * (0.02 L) * (168.11 g/mol) ≈ 0.01 g. Similarly, the molar mass of l-tyrosine is approximately 181.19 g/mol 3. For a 3 mM solution in 20 ml, the required mass is (0.003 mol/L) * (0.02 L) * (181.19 g/mol) ≈ 0.011 g. The initial recipe provided by the user indicated 0.5 g each of uric acid and l-tyrosine in 20 ml, which would result in much higher concentrations (approximately 148.7 mM and 138 mM, respectively). The procedure below assumes the intention was to achieve 3 mM concentrations, requiring the adjusted weights.

**Procedure:**

1. **Weighing of Solid Components:** Accurately weigh out 20 g of agar, 10 g of dried *Chlorella* algae extract, 15 g of yeast extract (powdered, for bacteriology), 3 g of potassium phosphate dibasic (K_2_HPO_4_), and 1 g of sodium pyruvate.
2. **Preparation of Uric Acid Solution (3** **mM):** Weigh approximately 0.01 g of uric acid (C_5_H_4_N_4_O_3_) and add it to 20 ml of distilled water. Uric acid has limited solubility in water (around 60 mg/L at 20 °C). To facilitate dissolution, gently heat the solution to around 37 °C and/or carefully add sterile 1 N NaOH dropwise while stirring until the uric acid is completely dissolved. Monitor the pH to avoid excessive alkalinity.
3. **Preparation of l-Tyrosine Solution (3** **mM):** Weigh approximately 0.011 g of l-tyrosine and add it to 20 ml of distilled water. l-tyrosine also exhibits low solubility in neutral pH water (around 0.45 g/L).
4. **Mixing and Autoclaving of Basal Medium:** Combine the weighed agar, *Chlorella* algae extract, yeast extract, potassium phosphate dibasic, and sodium pyruvate in a suitable autoclavable container with a capacity of at least 2 liters to accommodate potential foaming during autoclaving. Add 920 ml of distilled water and mix thoroughly to ensure the solid components are well dispersed. Autoclave the mixture at 121 °C for 15 min.
5. **Preparation and Addition of Glucose Solution (10** **%):** While the basal medium is autoclaving, prepare a 10 % glucose solution by dissolving 2 g of glucose in 18 ml of distilled water. Sterilize this solution by passing it through a 0.22 µm sterile filter into a sterile container. Adding glucose after autoclaving helps to avoid the Maillard reaction, which can occur between sugars and amino acids (present in yeast extract) at high temperatures, potentially reducing nutrient availability and forming inhibitory compounds.
6. **Addition of Uric Acid and l-Tyrosine Solutions:** Once the autoclaved medium has cooled to approximately 50–60 °C, aseptically add the prepared 20 ml uric acid solution. Gently heat the 20 ml l-tyrosine solution to around 50–60 °C and stir until the l-tyrosine is completely dissolved. Add this warm solution to the medium.
7. **Final Mixing and Pouring:** Gently swirl the complete GPL medium to ensure all components are uniformly distributed. Under aseptic conditions, pour approximately 15–20 ml of the medium into each sterile Petri dish (standard 90 mm diameter). This volume typically yields an agar layer of about 3 mm thickness, suitable for microbial cultivation. One liter of medium will yield approximately 50–67 Petri dishes. Allow the poured plates to cool and solidify completely at room temperature before use or store them inverted at 4 °C to prevent condensation.

## Method validation

**Fig. 1:**
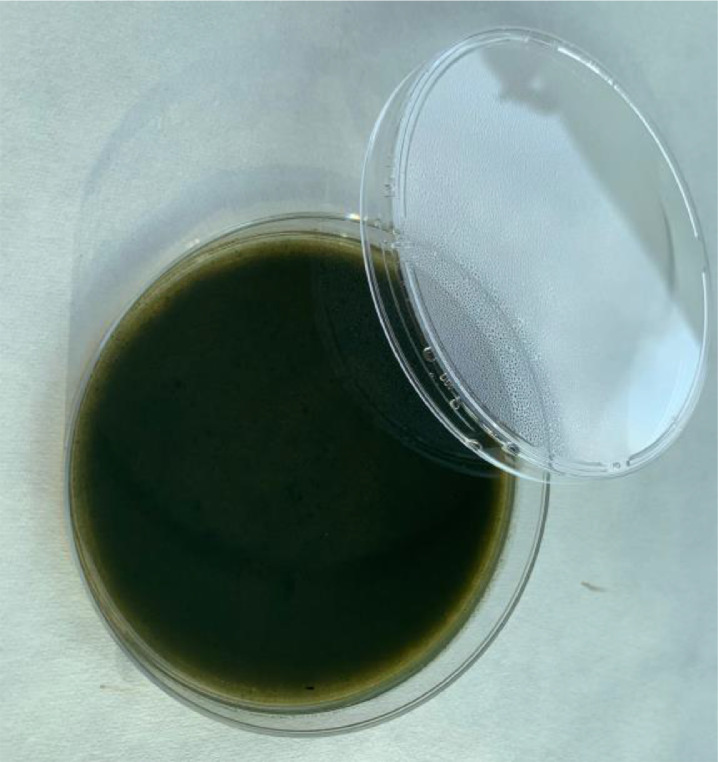
Petri dish with GPL medium.

The efficacy of the newly developed GPL medium in supporting the germination and growth of *P. larvae* was validated by comparing its performance to that of the standard MYPGP medium. Spores of *P. larvae* were inoculated onto both MYPGP agar and GPL agar plates Fig. 1, which were prepared according to the method described above. The plates were incubated at optimal temperatures for spore germination (35–37 °C).

**Fig. 2:**
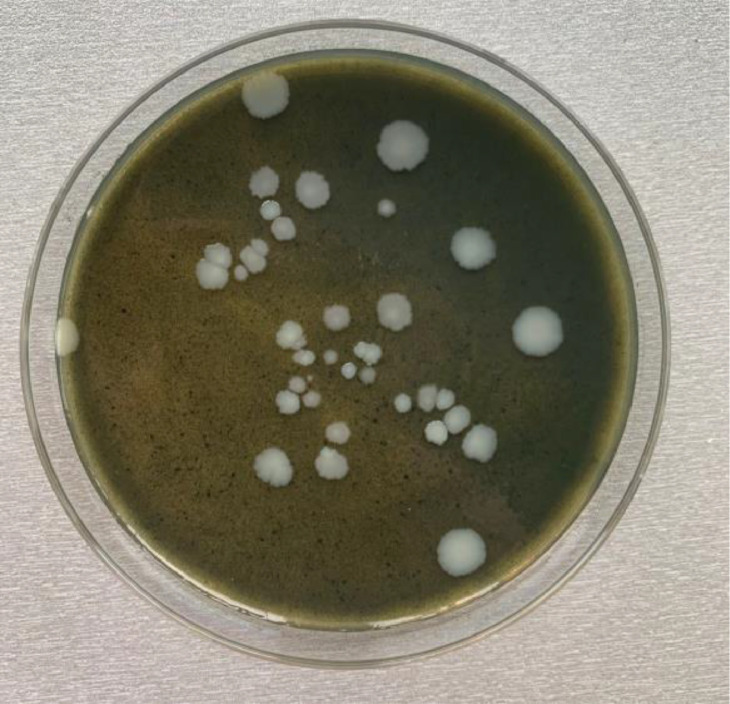
*Paenibacillus larvae* colony growth on a Petri dish containing GPL medium.

Following incubation, the extent of *P. larvae* colony growth and morphology on both media types was visually assessed. The bacterial cultures appeared macroscopically identical in terms of colony size, morphology, and density, indicating that the GPL medium supports the germination and proliferation of *P. larvae* spores equally as well as the conventional MYPGP medium (Fig. 2).

## Limitations

The current study validated the medium using a single reference strain of *Paenibacillus larvae* (CCM 4488). While its performance was comparable to the standard MYPGP medium, further studies should be conducted to investigate its applicability for a broader range of *P. larvae* genotypes (e.g., ERIC I-V). Evaluating the medium with various field isolates would be necessary to fully establish its versatility and confirm its diagnostic utility across different epidemiological contexts. Furthermore, the primary nutrient source, dehydrated *Chlorella*, may exhibit lot-to-lot variability. While our results demonstrate the efficacy of the specified batch, we recommend that laboratories implementing this method perform a small-scale quality control check (e.g., by comparing growth with a reference medium) when using a new batch of algae extract to ensure consistent performance..

## Related research article

None.

## Ethics statements

None.

## CRediT author statement

**Štěpán Ryba**: Conceptualization, Methodology, Writing- Original draft preparation. **Adéla Píchová**: Validity tests, Methodology. **Petr Mráz**: Writing- Original draft preparation, Investigation. **Irena Hoštičková**: Supervision. **Michaela Horčičková**: Supervision, Writing- Reviewing and Editing.

## Supplementary material *and/or* additional information [optional]

None.

## Declaration of competing interest

The authors declare that they have no known competing financial interests or personal relationships that could have appeared to influence the work reported in this paper.

## Data Availability

No data was used for the research described in the article.
